# LC-UV Determination of Baicalin in Rabbit Plasma and Tissues for Application in Pharmacokinetics and Tissue Distribution Studies of Baicalin after Intravenous Administration of Liposomal and Injectable Formulations

**DOI:** 10.3390/molecules21040444

**Published:** 2016-04-19

**Authors:** Yumeng Wei, Chao Pi, Gang Yang, Xiaoming Xiong, Yongshu Lan, Hongru Yang, Yang Zhou, Yun Ye, Yonggen Zou, Wenwu Zheng, Ling Zhao

**Affiliations:** 1 Department of Pharmaceutical Sciences, School of Pharmacy, Southwest Medical University, No. 3-319, Zhongshan Road, Jiangyang District, Luzhou 646000, China; weiyumeng-268@163.com (Y.W.); pichao2016@sina.com (C.P.); zhouyg2014@sina.com (Y.Z.); zl2006998@126.com (Y.Y.); 2 Department of Chemistry, the Institute of Basic Medical Sciences, Southwest Medical University, No. 3-319, Zhongshan Road, Jiangyang District, Luzhou 646000, China; gangy405@163.com; 3 Department of Pathology, the Affiliated Hospital of Southwest Medical University, No. 25, Taiping Street, Luzhou 646000, China; zl2006998@sina.com; 4 Department of Radiology, the Affiliated Hospital of Southwest Medical University, No. 25, Taiping Street, Luzhou 646000, China; LYBLUE2008@aliyun.com; 5 Department of Oncology, the Affiliated Hospital of Southwest Medical University, No. 25, Taiping Street, Luzhou 646000, China; yanghr2015@sina.com; 6 Department of Pharmacy, the Affiliated Hospital of Southwest Medical University, No. 25, Taiping Street, Luzhou 646000, China; 7 Department of Orthopedics, the Affiliated Hospital of Traditional Chinese Medicine of Southwest Medical University, No. 16, Chunhui Road, Longma Tan District, Luzhou 646000, China; zouyg2015@sina.com; 8 Department of Cardiovascular Medicine, The Affiliated Hospital of Southwest Medical University, No. 25, Taiping Street, Luzhou 646000, China; weilivipcqmc@163.com

**Keywords:** pharmacokinetics, tissue distribution, LC-UV, lung targeting, baicalin

## Abstract

A simple and sensitive LC-UV method to investigate the pharmacokinetics and biodistribution pattern of baicalin in rabbits was established and validated. Baicalin and the internal standard, rutin, were extracted from biosamples using acetonitrile as protein precipitation after pretreated with ammonium acetate buffer (pH 3.5; 1 M) to obtain a pure chromatographic peak and high extraction recovery. Chromatographic separation was achieved on a reverse-phase C18 column with a gradient elution at flow rate of 1.0 mL/min. UV absorption was set at 278 nm. Chromatographic response was linear over the ranges of 0.05–10.00 μg/mL in plasma and 0.05–300.00 μg/g in tissues with the limits of quantification of 50.0 ng/mL in plasma and tissues, and the limit of detection of baicalin in bio-samples of 15 ng/mL. The RSD of intra-and inter-day for the biosamples were from 4.19% to 10.84% and from 4.37% to 10.93%, respectively. The accuracy of plasma and tissue samples ranged from 81.6% to 95.2% and 80.8% to 98.4%, respectively. The extraction recoveries ranged from 81.5% to 88.3% for plasma, from 73.1% to 93.2% for tissues, respectively. Baicalin was stable in rabbit biosamples. The validated method was successfully applied to the study of the pharmacokinetics and tissue distribution of baicalin after intravenous administration of liposomal and injectable formulations to rabbits. Compared to baicalin injection, the pharmacokinetics and biodistribution behavior of baicalin was altered significantly in rabbits treated with its liposomes and drug concentration in the lungs was greatly increased.

## 1. Introduction

**Figure 1:**
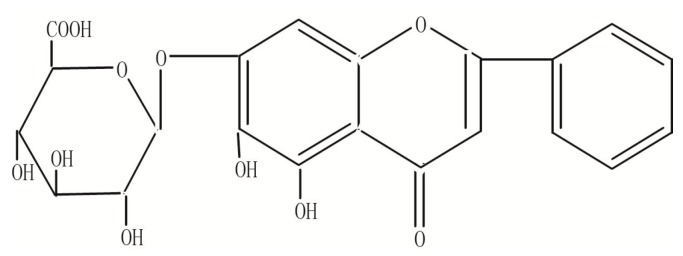
Chemical structure of baicalin.

Baicalin (7-glucuronic acid-5,6-dihydroxy-flavone, Figure 1), as one of the main bioactive flavone compounds, was isolated from the *Radix Scutellariae* that is a well-known traditional Chinese medicine used to treat diarrhea, fever, inflammatory and hepatic disease, and so on, in many countries [1]. Recently, a great deal of studies regarding baicalin have presented its excellent pharmacological anti-inflammation [2], anti-oxidation [3], anti-bacterial and anti-tumor activities [4,5]. At present, baicalin was widely used in the clinic either as a single compound such as its tablets and capsules or as a main active component in more than 40 kinds of preparations recorded in the Chinese Pharmacopeia (2015). However, the further development of baicalin was limited due to its low hydrophilicity, which has serious effects on the clinical use [6]. Many attempts have been made to develop new drug delivery systems for baicalin, such as inclusion complexes [7], nanoemulsions [8], solid lipid nanoparticles [9], phospholipid complexes and polyvinylpyrrolidone co-precipitate in order to improve the oral bioavailability [10,11,12]. Although these drug delivery systems were all used to increase its oral bioavailability to some extent, there are still some difficulties in meeting the requirements for clinical application and industrial production.

A recent study of baicalin indicated that it could inhibit proliferation of human lung carcinoma A549 and mouse Lewis lung cancer (LLC) in a dose- and time-dependent manner. It also could suppress tumor growth and prolong survival in C57BL/6 mice bearing LLC tumor and nude mice bearing A549 carcinoma [13]. Moreover, baicalin also could relieve lung injuries related to pancreatitis to some extent [14]. This made it necessary for us to develop a lung-targeting drug delivery system for baicalin. Interestingly, in the preliminary experiments, baicalin loaded liposomes prepared by an effervescent technique displayed lung targeting effects for the first time. This made it necessary to establish a simple and sensitive analytical method for the determination of baicalin in plasma and tissues to characterize its pharmacokinetics and tissue distribution patterns. The existing *in vivo* analytical methods for baicalin in plasma, serum, urine had been well developed mainly using the techniques of LC-UV [15], LC-MS [16], LC-DAD [17], UPLC-MS and enzyme-linked immunosorbent assay (icELISA) [18,19]. However, to date, to the best of our knowledge, the pharmacokinetics and tissue distribution of baicalin in rabbits via intravenous administration of its liposomal formulations have not yet been reported in the literature.

The objective of this study was thus to establish and validate for the first time a simple and sensitive LC-UV method to determine baicalin in rabbit plasma and tissue samples. The analytical method was applied successfully to investigate pharmacokinetics and tissue distribution characteristics of baicalin after a single intravenous administration of liposomal and injectable formulation to rabbits. This study was conducted to help design rational new dosage form of baicalin and evaluate its clinical effect.

## 2. Results and Discussion

### 2.1. Characteristics of Baicalin Loaded Liposomes

The freeze dried powders or granules of baicalin loaded liposomes were redissolved with ultrapure water to obtain a transparent and yellow solution. Particle size and PDI of baicalin-loaded liposomes measured by a NanoBrook 90 Plus Zeta instrument (Brookhaven Instruments, Holtsville, NY, USA) were 131.7 ± 11.7 nm and 0.19 ± 0.02, respectively. The entrapment efficiency of baicalin- loaded liposomes was 82.8% ± 1.24%, which was higher than the values (34.62%–60.11%) reported in the literature [20,21]. It was well known that there are significant differences in entrapment efficiency of liposomes prepared using different formulations and preparation technologies.

### 2.2. Optimization of Chromatographic Condition

According to several reports, a LC-UV method for the determination of baicalin in plasma was established using a reverse-phase column and various ratios of organic phases including acetonitrile or methanol and an aqueous phase including 0.2% (*v*/*v*) phosphoric acid or 0.1% (*v*/*v*) aqueous formic acid [22,23]. In preliminary experiments, these mobile phases were tried but acceptable peak symmetry and theoretical plates were not obtained. The acid concentration in the aqueous phase and the volumetric ratio of acetonitrile and methanol were found to significantly affect the peak symmetry and theoretical plates. In order to obtain symmetrical peaks and the best resolution of baicalin from endogenous compounds, various acid concentrations in the aqueous phase and ratios of acetonitrile and methanol were tested and optimized. As a result, a final mobile phase consisting of 1:1 (*v*/*v*) mixture of methanol and acetonitrile (A) and 0.4% (*v*/*v*) aqueous phosphoric acid (B) using a gradient elution of 85% B at 0–1 min, 85%–30% B at 1–14 min, 30%–85% B at 14–15 min was used to obtain satisfactory results in this study.

### 2.3. Assay Validation

#### 2.3.1. Selectivity

**Figure 2:**
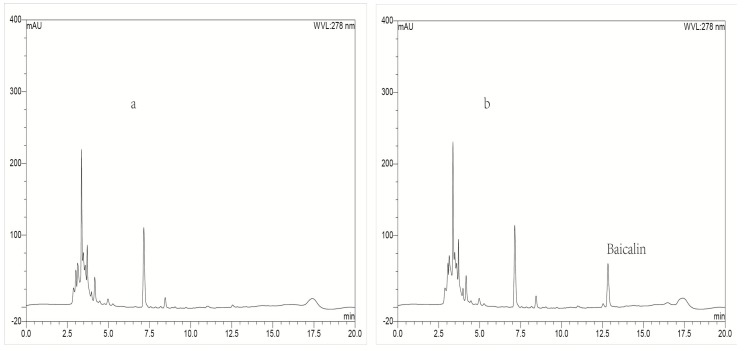

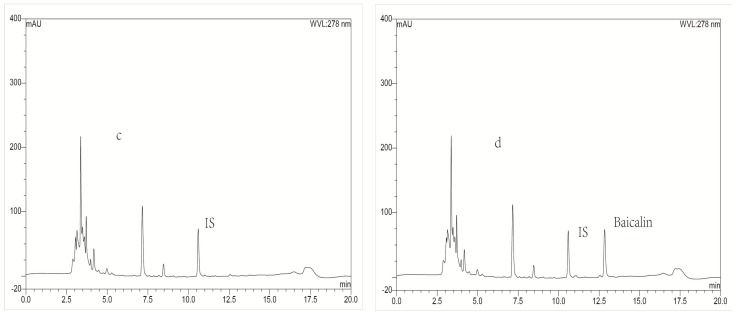
Representative LC-UV chromatograms obtained for blank plasma (**a**); blank plasma spiked with baicalin (**b**); blank plasma spiked with IS (**c**) and plasma biosample collected from a rabbit at 0.25 h after intravenous administration of baicalin-loaded liposomes at a dose of 10 mg/kg + IS (**d**).

**Figure 3:**
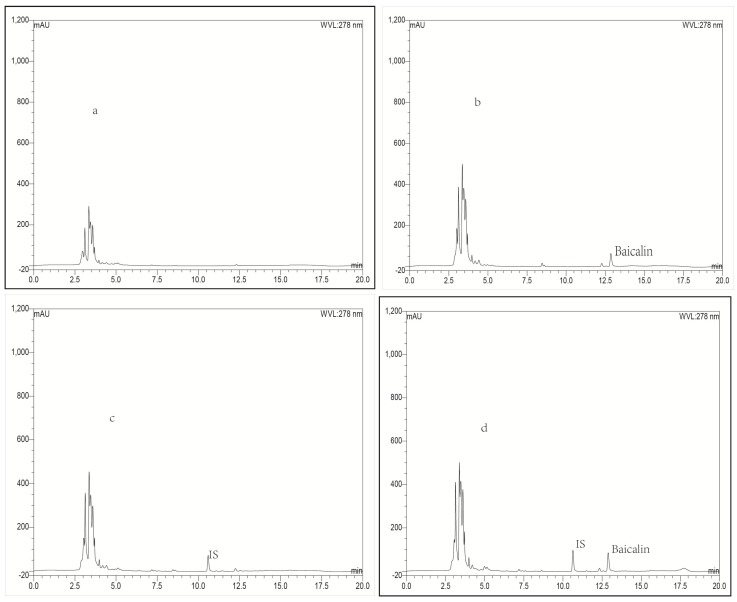
Representative LC-UV chromatograms obtained for blank lung tissue (**a**); blank lung tissue spiked with baicalin (**b**); blank lung tissue spiked with IS (**c**) and lung biosample collected from a rabbit at 0.25 h after intravenous administration of baicalin-loaded liposomes at a dose of 10 mg/kg + IS (**d**).

**Figure 4:**
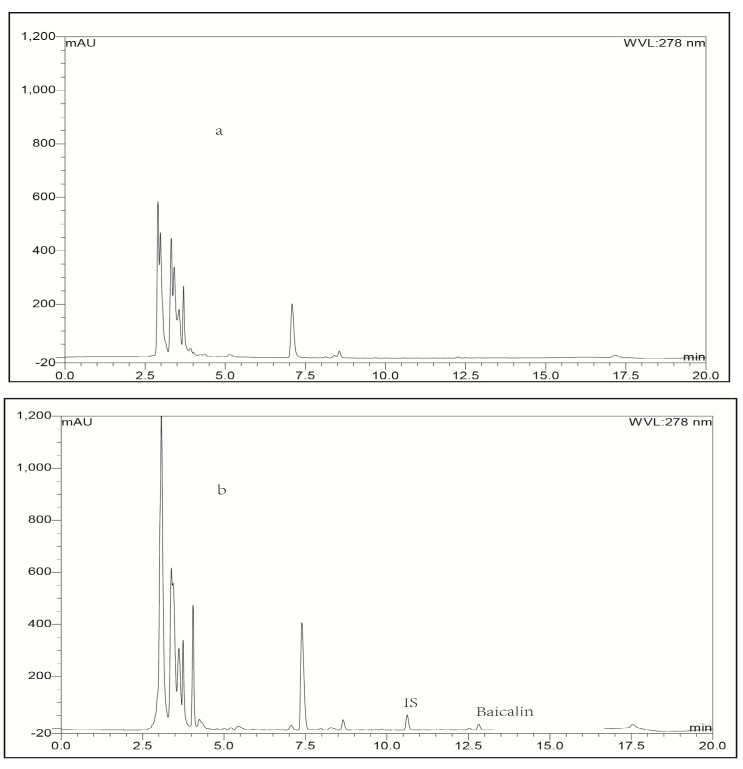
Representative LC-UV chromatograms obtained for blank heart tissue (**a**) and heart bio-sample collected from a rabbit at 0.25 h after intravenous administration of baicalin-loaded liposomes at a dose of 10 mg/kg + IS (**b**).

**Figure 5:**
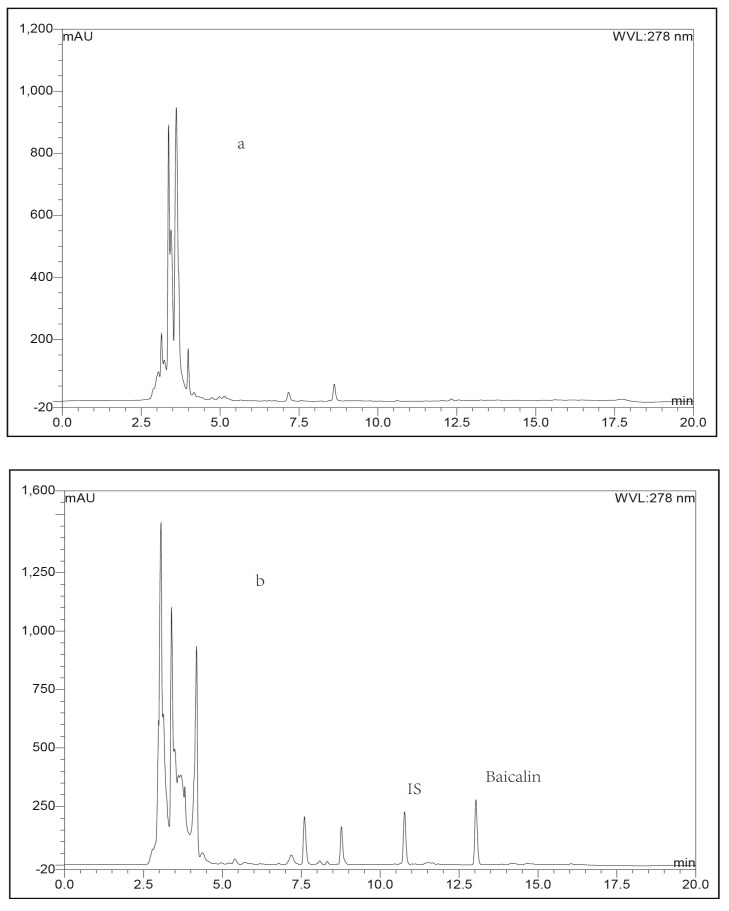
Representative LC-UV chromatograms obtained for blank liver tissue (**a**) and liver bio-sample collected from a rabbit at 0.25 h after intravenous administration of baicalin-loaded liposomes at a dose of 10 mg/kg + IS (**b**).

**Figure 6:**
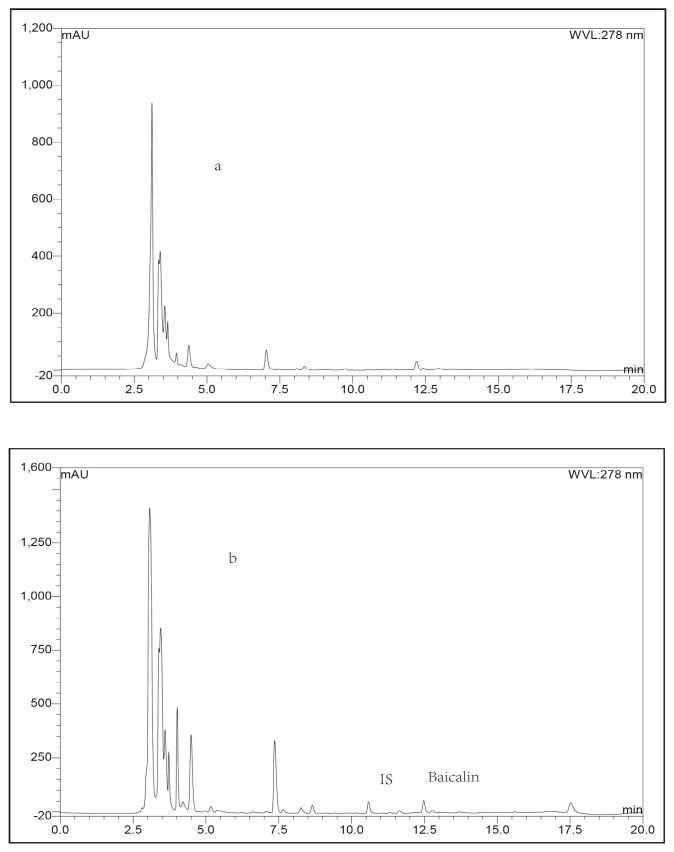
Representative LC-UV chromatograms obtained for blank spleen tissue (**a**) and spleen bio-sample collected from a rabbit at 0.25 h after intravenous administration of baicalin-loaded liposomes at a dose of 10 mg/kg + IS (**b**).

**Figure 7:**
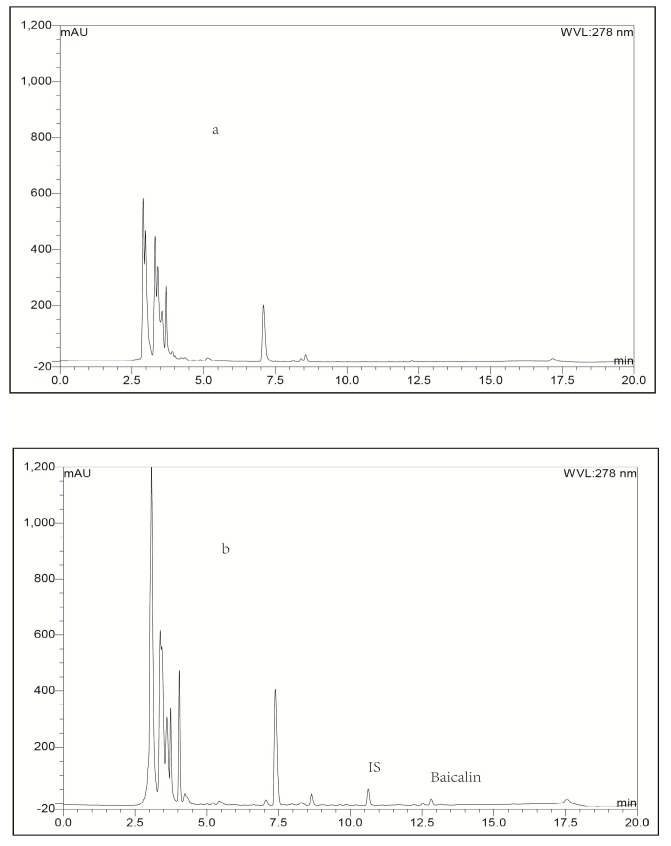
Representative LC-UV chromatograms obtained for blank kidney tissue (**a**) and kidney bio-sample collected from a rabbit at 0.25 h after intravenous administration of baicalin-loaded liposomes at a dose of 10 mg/kg + IS (**b**).

**Figure 8:**
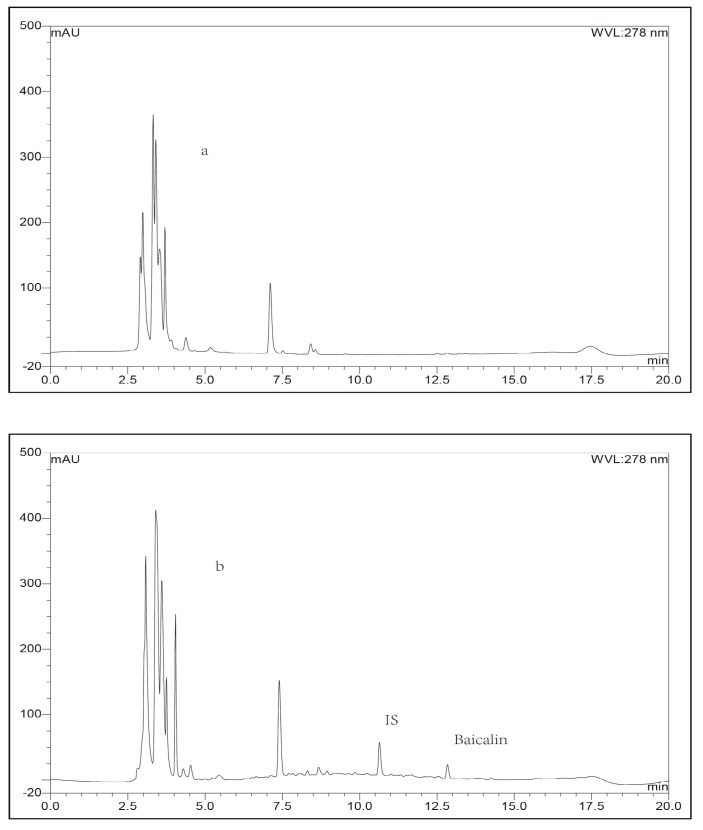
Representative LC-UV chromatograms obtained for blank stomach tissue (**a**) and stomach bio-sample collected from a rabbit at 0.25 h after intravenous administration of baicalin-loaded liposomes at a dose of 10 mg/kg + IS (**b**).

**Figure 9:**
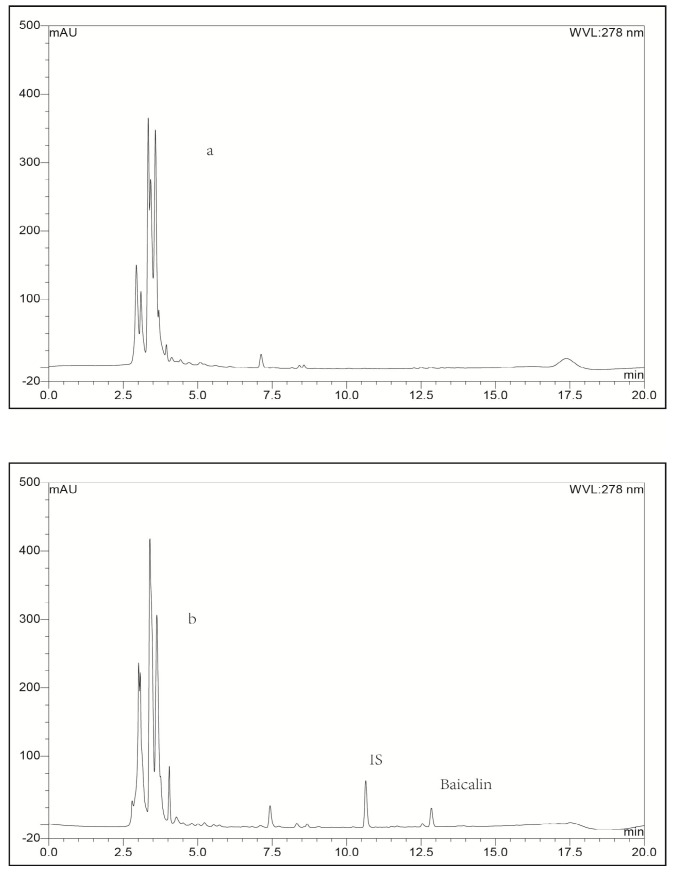
Representative LC-UV chromatograms obtained for blank brain tissue (**a**) and brain bio-sample collected from a rabbit at 0.25 h after intravenous administration of baicalin-loaded liposomes at a dose of 10 mg/kg + IS (**b**).

Typical chromatograms obtained with blank plasma or blank lung tissue homogenate as representative samples, blank plasma or blank lung homogenate spiked with baicalin or IS, and a plasma or lung tissue sample collected at 0.25 h spiked with IS after intravenous administration of baicalin loaded liposomes were shown in Figure 2 and Figure 3, respectively. To compare the chromatograms of samples from the other organs including heart, liver, spleen, kidney, brain and stomach, typical chromatograms obtained with blank tissue homogenates and tissue samples are shown in Figure 4, Figure 5, Figure 6, Figure 7, Figure 8 and Figure 9, respectively. It can be seen that a well-resolved baicalin peak free of interferences from endogenous compounds in rabbit plasma and tissues, was obtained, with a retention time of 12.8 min, which was shorter than previous results which ranged from 13.4 to 14.3 min [12,23,24]. In addition, the retention time of the IS was 10.4 min, which was obviously well separated from the peak of baicalin. Therefore, The LC-UV analytical method had good selectivity.

#### 2.3.2. Linearity Range, Limit of Quantification and Limit of Detection

Calibration curves of the peak area ratio of baicalin and IS (R) *versus* drug concentration (C) in plasma and tissues were plotted and the linear range and correlation coefficient (r) are presented in Table 1. The results were found to be linear within the ranges of 0.05–10 μg/mL for plasma, 0.05–10 μg/g for heart, brain and spleen, 0.2–10 μg/g for kidney, 0.05–5 μg/g for stomach, 0.2–5 μg/g for liver, 0.75–300 μg/g for lung, (r > 0.999). The assay method offered limits of quantification (LOQ) of 50.0 ng/mL in plasma and 50.0 ng/g in tissue homogenate samples, and the limit of detection (LOD) of baicalin in plasma and tissue samples were 15 ng/mL and 15 ng/g, respectively, which were lower than the results reported in [23]. This suggested that this LC-UV analytical method is sensitive enough for study of the pharmacokinetic and tissue distribution behavior of baicalin in rabbits.

#### 2.3.3. Accuracy and Precision

Both the accuracy and precision of analysis method were determined by using three QC samples at low, medium and high concentrations. Besides, the precision needed to be calculated the relative standard deviation (RSD) and the results were shown in Table 2. The RSD values of intra-and inter-day for the plasma and tissues were from 4.19 to 10.84 and from 4.37 to 10.93, respectively, which were less than 15, revealed that the precision of the analytical method was high.

The accuracy of plasma and tissue samples ranged from 81.6% to 95.2% and 80.8% to 98.4%, respectively, which indicated that there was no interference from endogenous components.

#### 2.3.4. Extraction Recovery

Baicalin is a flavanoid glycoside with relatively high polarity and poor solubility. Importantly, it has characteristics of easy biological degradation and temperature- and pH-dependent stability. Several studies reported in the literature have indicated that it is necessary to maintain a stable acidic environment in biosamples for the processing and storage of baicalin [25,26]. At present, the pretreatment methods of baicalin in plasma, urine and serum mainly include solid-phase extraction (SPE), protein precipitation (PPT) and liquid-liquid extraction (LLE) [22]. Because baicalin is an unstable compound, the relatively long column activation time, sample loading time and elution time of SPE resulted in its degradation in the biosamples to some extent. Although PPT has high extraction recovery for many compounds and easy sample manipulation, the absolute recovery of baicalin obtained from rat plasma by the PPT pretreatment method was found to be only 66.1% [23]. Among these pretreatment methods, LLE using ethyl acetate or acetone as extraction regent was the most frequently used method to process baicalin biosamples with absolute recoveries ranging from 60% to 70% [27,28]. In the preliminary study, the LLE method reported above was used, but it led to low extraction recovery (<60%) in rabbit tissues and the samples contained endogenous substances which influenced with the determination of baicalin and IS in rabbit biosamples. According to [29], on the basis of LLE, the use of inorganic salts may both significantly increase the extraction efficiency of the drug from aqueous phase and further purify the biosamples. Therefore, in the present study, the effect of pH values (3.5, 4.5 and 5.5) and concentration (0.5 M, 1 M and 2 M) of ammonium acetate buffer as inorganic salt on the extraction recovery of baicalin in biosamples was investigated using the same volume of acetonitrile as in the protein precipitation. The results showed that the optimal pretreatment method composed of 250 µL of ammonium acetate buffer (pH 3.5; 1 M) and 3 mL of acetonitrile could not only afford high extraction recoveries of baicalin in biosamples that ranged from 81.5% to 88.3% for plasma, from 73.1% to 93.2% for tissues, respectively (Table 3), but also remove the interfering substances in plasma or tissue samples (Figure 2, Figure 3, Figure 4, Figure 5, Figure 6, Figure 7, Figure 8 and Figure 9).

#### 2.3.5. Stability

The stability of baicalin in rabbit plasma or tissues with QC samples was investigated and the results are presented in Table 4. The relative error (RE) of baicalin between the initial concentrations and the concentration following five freeze-thaw cycles ranged from −14.7% to 7.0%, at −20 °C for 15 days ranged from −14.3% to −4.1%, and post-preparative stability after 12 h ranged from −14.3% to 4.8%, respectively, which were less than 15%. Hence, it suggested that baicalin was stable in rabbit plasma and tissues.

### 2.4. Pharmacokinetics and Tissue Distribution Study

#### 2.4.1. Plasma Pharmacokinetics Study

**Figure 10:**
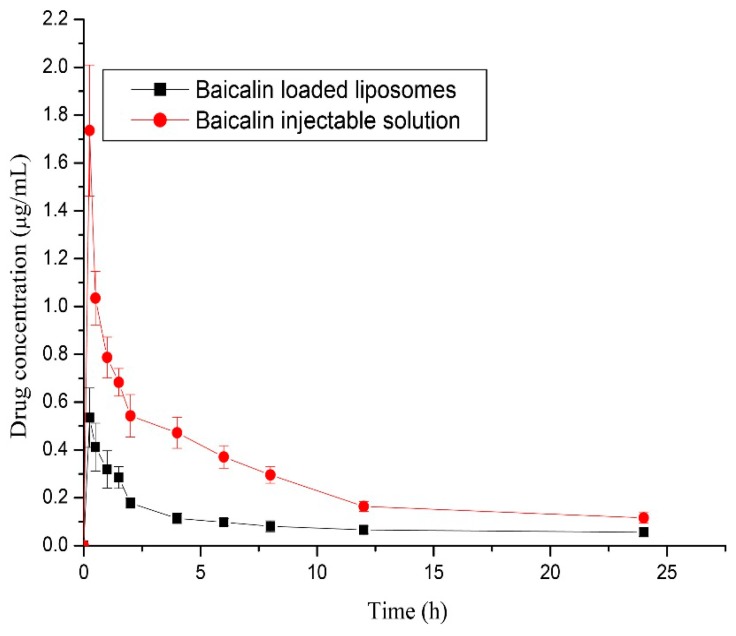
Mean plasma drug concentration-time profile of baicalin determined by LC-UV method via intravenous administration of baicalin loaded liposomes and its injectable solution at a dose of 10 mg/kg to rabbits. Each point represents the mean + SD (*n* = 5).

The LC-UV analytical method developed in this study was used to determine the plasma concentration of baicalin in rabbits after a single intravenous administration of baicalin-loaded liposomes and its injectable solution at a dose of 10 mg/kg to rabbits. The mean plasma drug concentration-time profile and the main pharmacokinetic parameters are shown in Figure 10 and Table 5, respectively. It can be seen from the plasma drug concentration-time profile that the drug concentration in plasma decreased rapidly and was detectable up to at least 24 h. As far as pharmacokinetic parameters, the distribution phase (t_1/2α_) half-lifes was 0.038 h and the half-life of the elimination phase (t_1/2β_) was 1.02 h after intravenous administration of baicalin-loaded liposomes to rabbits, which were shorter than those of its injectable solution (0.141 h and 3.88 h, respectively). The plasma clearance (CL_z_) and the volume of distribution (V_z_) of baicalin-loaded liposomes (3.51 L/h/kg and 51.77 L/kg) were significantly higher than those of its injectable solution (1.34 L/h/kg and 10.27 L/kg).

The area under the curve (AUC_(0−24h)_) of the liposomal formulation was about 3-fold lower than that of injectable formulation, suggesting that the liposome carrier could increase the biodistribution of baicalin in tissues.

#### 2.4.2. Tissue Distribution Study

**Figure 11:**
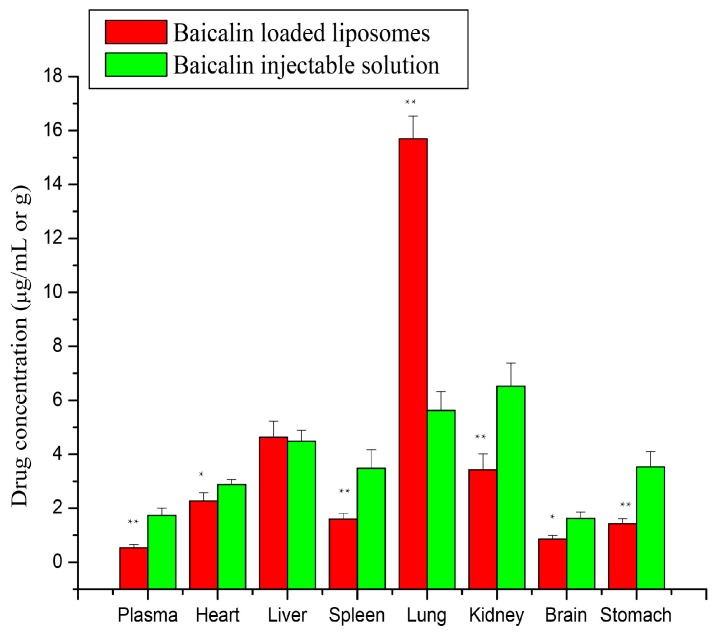
Distribution in plasma and tissues after via intravenous administration of baicalin loaded liposomes and its injectable solution at a dose of 10 mg/kg to rabbits. Each point represents the mean + SD (*n* = 5). ** *p* < 0.01 compared with baicalin injection; * *p* < 0.05 compared with baicalin injection.

The drug concentration in plasma and tissues was determined by the developed LC-UV analytical method and the biodistribution behavior of baicalin at 0.25 h after a single intravenous administration of baicalin-loaded liposomes and its injectable solution is presented in Figure 11. It can be seen from Figure 11 that the liposome carrier significantly altered the tissue distribution characteristics of baicalin in rabbits in comparison with its injectable solution. After intravenous administration of liposomal baicalin, the drug concentration in the lungs was the highest (15.693 ± 0.839 µg/g) among all tissues or plasma, that is, the drug concentration decreased in main organ tissues in the following order: lung > liver > kidney > spleen. When administered with its injectable solution, drug concentration decreased in the main organ tissues in the following order: kidney > lung > liver > spleen. Baicalin concentration in the lungs after intravenous administration of baicalin-loaded liposomes was increased from 5.627 ± 0.697 µg/g to 15.693 ± 0.839 µg/g (2.789-fold) in comparison with its injectable solution.

From the clinical point of view, the biodistribution of the liposomes is an important parameter. The biodistribution behavior of the liposome carrier mainly depends on its physiochemical characterization including particle size, zeta potential, membrane lipid component and steric stabilization [30]. Many studies have indicated that liposomes mostly accumulate in the organs of the reticuloendothelial system such as liver, spleen and lung within the first 15–30 min after intravenous administration of a liposomal formulation [31]. Therefore, it demonstrated that the liposome carrier could deliver baicalin mainly into lung tissue after intravenous administration.

## 3. Materials and Methods

### 3.1. Materials and Animals

The crude baicalin was obtained from Xinxiang Bokai Bio-Technology Co., Ltd. (Henan, China). The baicalin reference sample used in the analysis was provided by the National Institute for Food and Drug Control of China (Beijing, China). The internal standard rutin was obtained from Chengdu Mansite Pharmaceutical Co., Ltd. (Sichuan, China). Phospholipon 90H (HSPC) was obtained from Shanghai Toshisun Bio-Technology Co., Ltd. (Shanghai, China). Citric acid (injection grade), carbonic acid monosodium salt (NaHCO_3_), Tween-80 (injection grade), anhydrous alcohol and ammonium acetate were purchased from Luzhou Juhe Chemical Co., Ltd., (Luzhou, China). Acetonitrile and methanol (HPLC grade) were obtained from Chengdu Jinghong Co., Ltd., (Chengdu, China). 0.9% Sodium chloride injection was supplied from the First Affiliated Hospital of Southwest Medical University (Luzhou, China). Ultrapure water used in this study was prepared in our experiments. New Zealand white rabbits (weight 1.5–2.0 kg) were obtained from the Laboratory Animal Center of Southwest Medical University (Luzhou, China). The rabbits were maintained in a temperature- and moisture-controlled (20 ± 2 °C and 55% ± 10%, respectively) room with a 12 h light-dark cycle, and allowed free access to food and water except when fasted for 12 h before experiments. The experimental protocol was approved by the Southwest Medical University Animal Ethical Experimentation Committee (No: 2013002).

### 3.2. Preparation of Baicalin Lposomes

Baicalin-loaded liposomes used in this study were prepared by the effervescent dispersion technique. Briefly, baicalin (100 mg), HSPC (100 mg), Tween-80 (50 mg) and citric acid (50 mg) were weighed and dissolved in an appropriate volume of ethanol. The solution was added dropwise to aqueous NaHCO_3_ solution (0.5%, *w*/*v*) containing mannitol (5.0%, *w*/*v*) at 15–20 °C with continuous stirring. The mixture solution was continually stirred until it became cheese-like. Finally the cheese-like products were lyophilized and stored at 2–8 °C for further study.

### 3.3. Equipment and LC-UV Conditions

The LC-UV system used for determination of baicalin concentration in rabbit plasma and tissues in this study was an Ultimate 3000 series chromatographic system (Dionex, MA, USA) equipped with an Inertsil ODS-SP reverse-phase C18 column (4.6 × 250 mm, 5 µm) protected by a Phenomenex C18 guard column (4.0 mm × 3.0 mm, 5 µm) (Torrance, CA, USA) with the column temperature maintained at 35 °C. UV absorption was set at 278 nm. The mobile phase consisted of the mixture of methanol and acetonitrile (1:1, *v*/*v*) (A) and 0.4% (*v*/*v*) aqueous phosphoric acid (B). The gradient began with 85% eluent B for 1 min, decreased linearly to 30% eluent B for 13 min. The gradient was then returned to initial condition for 1 min and was maintained in this condition for 1 min. The flow rate was 1.0 mL/min and 20 µL of sample solution was injected into LC-UV system.

### 3.4. Preparation of Calibration Standards and Quality Control Samples

Blood and tissue samples were obtained from experimental rabbits and stored at −20 °C until baicalin was assayed. 0.5 mL of blank plasma or tissue homogenate suspension was mixed with 50 µL of various known concentrations of baicalin standard working solutions and 50 µL of rutin solution (80.0 µg/mL in methanol) as the internal standard to obtain a series of baicalin standard solutions at concentrations ranged from 0.05 to 10 μg/mL for plasma, from 0.05 to 10 μg/g for heart, brain and spleen, from 0.2 to 10 μg/g for kidney, from 0.05 to 5 μg/g for stomach, from 0.2 to 5 μg/g for liver and from 0.75 to 300 μg/g for lung. Quality control (QC) samples were prepared by blank plasma or blank tissue homogenate suspension at low, medium and high baicalin concentrations.

### 3.5. Validation of Analysis Method

In order to confirm the capability of the method to satisfy the requirements of the application, the analytical method was validated in terms of the selectivity, linearity, precision, accuracy, extraction recovery and stability according to the bioanalytical method validation guidance from the U.S. Food and Drug Administration (2001 version).

The selectivity of the method was determined by comparing the chromatography of blank biosamples, blank biosamples spiked with baicalin and (or) IS, and biosamples following intravenous administration of baicalin-loaded liposomes to ensure no interference. Because quantitative analytical results are greatly affected by the quality of the calibration curve, seven different baicalin concentrations with the fixed IS concentration in blank rabbits plasma or tissue homogenate suspension were used in this study and the standard curves were obtained by plotting the peak area ratios (R) of baicalin and IS against the corresponding baicalin concentration (C). Intra-day and inter-day accuracy and precision were investigated by determining QC samples at low, medium and high concentration on the same day and on three separate days. The extraction recoveries of baicalin at three QC concentrations were calculated by comparing the peak area of baicalin in extracted biosamples with those obtained by directly determining baicalin standard solutions at the same concentration. The stability of baicalin in rabbit plasma and tissue samples was investigated at three QC concentrations. For long-term stability, biosamples were kept at −20 °C for 15 days; freeze-thaw stability was tested through five freeze-thaw cycles on samples that were frozen at −20 °C and thawed at room temperature on days 0, 2, 4, 6, 8; and post-preparative stability was investigated by reanalyzing QC samples at five time points for 12 h.

### 3.6. Application in Pharmacokinetics and Tissue Distribution Study

The developed LC-UV method was applied to quantify the plasma or tissue drug concentration of baicalin. For a pharmacokinetics study, baicalin-loaded liposomes and its injectable formulation were injected through the ear vein of rabbits at a dose of 10 mg baicalin per kg body weight. Blood samples (1.5 mL) were collected from the marginal ear vein into heparinized centrifuge tubes just before dosing (0 h) and after at 0.25, 0.5, 1, 1.5, 2, 4, 6, 8, 12 and 24 h. The blood samples were immediately centrifuged at 5000 rpm for 5 min to separate the plasma and stored at −20 °C until analysis.

For the tissue distribution study, baicalin-loaded liposomes and its injectable formulation were administered to five experimental rabbits intravenously with a single dose equivalent to 10 mg baicalin per kg body weight. The rabbits were sacrificed at 0.25 h after intravenous administration. Heart, liver, spleen, lung, kidney, stomach and brain were sampled immediately and washed with 0.9% saline solution. After surface water was dried, 2 g of tissue sample was homogenized with 4 mL methanol and stored at −20 °C until analysis for baicalin content.

Plasma or tissue homogenate samples (0.5 mL) were mixed with 50 µL of rutin internal standard solution containing IS (80.0 µg/mL in methanol) and 250 µL of ammonium acetate buffer (pH 3.5, 1 M) and vortexed for 3 min, and then 3 mL of acetonitrile was added to the solution by vortex mixing for 5 min. After centrifugation at 10,000 rpm for 10 min, the clear supernatant was collected and evaporated to dryness under a nitrogen gas stream at 40 °C. The dry sample was reconstituted with 200 µL of mobile phase and vortex-mixed, and centrifuged at 10,000 rpm for 10 min. Then 20 µL of the clear supernatant was injected into chromatographic system.

### 3.7. Data Analysis

The pharmacokinetic parameters including the area under the plasma drug concentration-time curve (AUC_0-t_), the half-lives of distribution phase (t_1/2α_), the half-lives of elimination phase (t1/2β), apparent volume of distribution (Vz), clearance rate (CLz) and the peak concentration (Cmax) were calculated by the pharmacokinetical software program DAS 2.0 (Mathematical Pharmacology Professional Committee of China, Shanghai, China). All results were expressed as the mean ± standard deviation (SD) and statistically significant difference between the pharmacokinetic data of baicalin loaded liposomes and its injection as control was evaluated using two-tailed t test. The level of significant difference was set at *p* < 0.05.

## 4. Conclusions

Baicalin and the internal standard, rutin were extracted from biosamples using acetonitrile as protein precipitation reagent after pretreatment with ammonium acetate buffer (pH 3.5, 1 M) to obtain chromatographically pure peaks and high extraction recovery. Therefore, the developed reverse-phase LC-UV analytical method for the determination of baicalin in rabbit plasma and tissues offers excellent selectivity, linearity, accuracy, precision, recovery and a short running time. The validated LC-UV assay method has been successfully applied to pharmacokinetic and tissue distribution studies of baicalin after intravenous administration of baicalin-loaded liposomes and its injectable solution to rabbits. In fact, the assay method is also suitable for quantitative determination of baicalin in biosamples in preclinical and clinical experimental phase studies of baicalin-loaded liposomes. Interestingly, after intravenous administration of baicalin-loaded liposomes, the liposome carrier significantly altered the pharmacokinetics and biodistribution behavior of baicalin in rabbits in comparison with its injectable solution and showed greatly increased drug concentrations in the lungs. It suggested that baicalin-loaded liposomes have good lung targeting characteristics and advantages for the treatment of lung diseases.

## Figures and Tables

**Table 1:** Linear ranges, standard curves and correlation coefficients of baicalin in different rabbit biosamples ^a^.

Biosample	Standard Curves	Linear Ranges (μg/g or μg/mL) ^b^	Correlation Coefficient (r)
Heart	A = 0.0967C + 0.0072	0.05–10	r = 0.9997
Kidney	A = 0.0866C + 0.0083	0.2–10	r = 0.9996
Brain	A = 0.0788C + 0.0071	0.05–10	r = 0.9997
Stomach	A = 0.0732C + 0.0304	0.05–5	r = 0.9994
Plasma	A = 0.1017C − 0.0037	0.05–10	r = 0.9995
Liver	A = 0.0657C + 0.0003	0.2–5	r = 0.9991
Spleen	A = 0.0584C + 0.0123	0.05–10	r = 0.9996
Lung	A = 0.059C + 0.2118	0.75–300	r = 0.9992

^a^ The values are arithmetic means, *n* = 3; ^b^ The unit of drug concentration in plasma: μg/mL; The unit of drug concentration in tissue: μg/g.

**Table 2:** Intra- and inter-day accuracy and precision data for baicalin in rabbit plasma and tissues ^a^.

Samples	Added Concentration ^b^	Intra-Day			Inter-Day		
		Measured Concentration ^b^	Accuracy (%)	(RSD) (%)	Measured Concentration ^b^	Accuracy (%)	(RSD) (%)
Plasma	0.0525	0.045	85.2	6.91	0.048	90.9	6.24
	4.7500	4.223	88.3	9.06	4.518	95.2	8.37
	9.5000	7.747	81.6	8.69	8.527	89.5	9.64
Heart	0.0525	0.044	83.8	4.42	0.046	87.4	4.37
	4.7500	4.015	84.5	9.65	4.346	91.5	9.94
	9.5000	8.912	93.8	6.75	7.681	80.6	7.47
Liver	0.210	0.191	91.3	6.52	0.183	87.2	6.97
	2.375	1.926	81.1	10.24	2.094	88.4	10.81
	4.750	3.837	80.7	9.57	4.025	84.7	9.18
Spleen	0.0525	0.047	88.5	7.97	0.048	90.4	7.24
	4.7500	4.126	86.6	5.46	4.388	92.7	5.02
	9.5000	8.005	84.2	9.11	7.732	81.3	9.48
Lung	0.8	0.663	82.9	4.19	0.649	80.8	4.74
	142.5	132.245	92.5	5.64	119.272	83.7	5.38
	285.0	232.275	81.4	8.84	251.655	88.6	9.17
Kidney	0.21	0.206	98.4	9.67	0.174	81.8	10.83
	4.75	4.273	89.9	5.14	3.993	84.3	5.73
	9.50	8.829	92.2	8.42	8.278	87.6	8.62
Stomach	0.0525	0.051	98.1	9.73	0.049	93.5	9.28
	2.3750	2.137	90.2	8.03	1.978	83.3	8.75
	4.7500	4.355	91.3	10.84	4.235	89.2	10.73
Brain	0.0525	0.049	95.1	10.64	0.044	80.9	10.93
	4.7500	3.959	83.4	7.99	4.088	86.4	7.63
	9.500	8.544	89.3	8.53	7.947	83.6	8.28

^a^ The values are arithmetic means, *n* = 3; ^b^ The unit of drug concentration in plasma: μg/mL; The unit of drug concentration in tissue: μg/g.

**Table 3:** Extraction recovery of baicalin in rabbit plasma and tissues ^a^.

Samples	Added Concentration (μg/mL or μg/g) ^b^	Extraction Recovery (μg/mL or μg/g) ^b^	RSD (%)
Plasma	0.0525	85.7	2.51
	4.75	88.3	9.28
	9.5	81.5	8.87
Heart	0.0525	87.9	1.08
	4.75	82.7	1.58
	9.5	86.9	7.14
Kidney	0.21	78.7	9.73
	4.75	93.2	9.51
	9.5	80.3	5.84
Brain	0.0525	81.7	3.38
	4.75	86.4	2.12
	9.5	79.2	3.03
Stomach	0.0525	87.2	9.89
	2.375	81.8	2.75
	4.75	94.6	6.68
Liver	0.21	92.3	8.16
	2.375	80.7	5.47
	4.75	73.1	8.58
Spleen	0.0525	91.9	9.35
	4.75	78.3	8.07
	9.5	81.5	3.21
Lung	0.8	93.2	1.68
	142.5	9.9	3.85
	285.0	87.1	8.65

^a^ The values are arithmetic means, *n* = 3; ^b^ The unit of drug concentration in plasma: μg/mL; The unit of drug concentration in tissues: μg/g.

**Table 4:** Stability of baicalin in rabbit plasma and tissues with QC samples determined by LC-UV ^a^.

		Freeze-Thaw Stability		Storage Stability		Post-Preparative Stability	
Samples	Added Concentration (μg/mL or μg/g) ^b^	Measured Concentration (μg/mL or μg/g) ^b^	Relative Error (%)	Measured Concentration (μg/mL or μg/g) ^b^	Relative Error (%)	Measured Concentration (μg/mL or μg/g) ^b^	Relative Error (%)
	0.0525	0.047	−10.5	0.049	−6.7	0.045	−14.3
Plasma	4.75	4.138	−12.9	4.258	−10.4	4.371	−8.0
	9.5	8.362	−12.0	8.139	−14.3	8.697	−8.5
	0.0525	0.048	−8.6	0.045	−14.3	0.047	−10.5
Heart	4.75	4.154	−12.5	4.123	13.2	4.472	−5.9
	9.5	8.118	−14.5	8.767	−7.7	8.93	−6.0
	0.21	0.18	−14.3	0.18	−14.3	0.19	−9.5
Kidney	4.75	4.164	−12.3	4.109	−13.5	4.497	−5.3
	9.5	8.569	−9.8	8.895	−6.4	9.154	−3.6
	0.0525	0.046	−12.4	0.048	−8.6	0.049	−6.7
Brain	4.75	4.272	−10.1	4.172	−12.2	4.625	−2.6
	9.5	8.763	−7.8	8.863	−6.7	9.29	−2.2
	0.0525	0.045	−14.3	0.048	−8.6	0.047	−10.5
Stomach	2.375	2.149	−9.5	2.218	−6.6	2.195	−7.6
	4.75	4.056	−14.6	4.476	−5.8	4.556	−4.1
	0.21	0.193	−8.1	0.182	−13.3	0.22	4.8
Liver	2.375	2.068	−12.9	2.122	−10.7	2.389	0.6
	4.75	4.054	−14.7	4.365	−8.1	4.891	3.0
	0.0525	0.046	−12.4	0.047	−10.5	0.053	1.0
Spleen	4.75	3.956	−16.7	4.556	−4.1	4.689	−1.3
	9.5	8.252	−13.1	8.589	−9.6	9.138	-3.8
	0.8	0.744	−7.0	0.72	−10.0	0.84	5.0
Lung	142.5	125.828	−11.7	130.28	−8.6	135.66	−4.8
	285	256.35	−10.1	265.79	−6.7	271.45	−4.8

^a^ The values are arithmetic means, *n* = 3; ^b^ The unit of drug concentration in plasma: μg/mL; The unit of drug concentration in tissues: μg/g.

**Table 5:** The comparative pharmacokinetic parameters via intravenous administration of baicalin loaded liposomes and its injectable solution at a dose of 10 mg/kg to rabbits. Each point represents the mean + SD (*n* = 5).

Samples	t_1/2α_ (h)	t_1/2β_	C_max_ (μg/mL)	AUC_(0–24h)_ (mg/L·h)	CLz (L/h/kg)	Vz (L/kg)
Baicalin liposomes	0.038	1.02	0.535 *	2.42 *	3.51	51.77
Baicalin injection	0.141	3.88	1.736	7.07	1.34	10.27

* *p* < 0.01 compared with baicalin injection.

## References

[1] Pan T.L., Wang P.W., Huang C.H., Leu Y.L., Wu T.H., Wu Y.R., You J.S. (2015). Herbal formula, Scutella-riae radix and Rhei rhizoma attenuate dimethylnitrosamine-induced liver fibrosis in a rat model. Sci. Rep..

[2] Cui L., Feng L., Zhang Z.H., Jia X.B. (2014). The anti-inflammation effect of baicalin on experimental colitis through inhibiting TLR4/NF-κB pathway activation. Int. Immunopharmacol..

[3] Wang S.C., Chen S.F., Lee Y.M., Chuang C.L., Bau D.T., Lin S.S. (2013). Baicalin scavenges reactive oxygen species and protects human keratinocytes against UVC-induced cytotoxicity. Vivo.

[4] Lixuan Z., Jingcheng D., Wenqin Y., Jianhua H., Baojun L., Xiaotao F. (2010). Baicalin attenuates inflammation by inhibiting NF-κB activation in cigarette smoke induced inflammatory models. Pulm. Pharmacol. Ther..

[5] Chan F.L., Choi H.L., Chen Z.Y., Chan P.S., Huang Y. (2000). Induction of apoptosis in prostate cancer cell lines by a flavonoid, baicalin. Cancer Lett..

[6] Srinivas N.R. (2010). Baicalin, an emerging multi-therapeutic agent: Pharmacodynamics, pharmacokinetics, and considerations from drug development perspectives. Xenobiotica.

[7] Li J., Zhang M., Chao J., Shuang S. (2009). Preparation and characterization of the inclusion complex of Baicalin (BG) with beta-CD and HP-β-CD in solution: An antioxidant ability study. Spectrochim. Acta A Mol. Biomol. Spectrosc..

[8] Zhao L., Wei Y., Huang Y., He B., Zhou Y., Fu J. (2013). Nanoemulsion improves the oral bioavailability of baicalin in rats: *In vitro* and *in vivo* evaluation. Int. J. Nanomed..

[9] Liu Z., Zhang L., He Q., Liu X., Okeke C.I., Tong L., Guo L., Yang H., Zhang Q., Zhao H. (2015). Effect of Baicalin-loaded PEGylated cationic solid lipid nanoparticles modified by OX26 antibody on regulating the levels of baicalin and amino acids during cerebral ischemia-reperfusion in rats. Int. J. Pharm..

[10] Li N., Je Y.J., Yang M., Jiang X.H., Ma J.H. (2011). Pharmacokinetics of baicalin-phospholipid complex in rat plasma and brain tissues after intranasal and intravenous administration. Pharmazie.

[11] Wu J., Chen D., Zhang R. (1999). Study on the bioavailability of baicalin-phospholipid complex by using HPLC. Biomed. Chromatogr..

[12] Li B., He M., Li W., Luo Z., Guo Y., Li Y., Zang C., Wang B., Li F., Li S. (2013). Dissolution and pharmacokinetics of baicalin-polyvinylpyrrolidone coprecipitate. J. Pharm. Pharmacol..

[13] Du G., Han G., Zhang S., Lin H., Wu X., Wang M., Ji L., Lu L., Yu L., Liang W. (2010). Baicalin sup-presses lung carcinoma and lung metastasis by SOD mimic and HIF-1alpha inhibition. Eur. J. Pharmacol..

[14] Li Z., Xia X., Zhang S., Zhang A., Bo W., Zhou R. (2009). Up-regulation of Toll-like receptor 4 was suppressed by emodin and baicalin in the setting of acute pancreatitis. Biomed. Pharmacother..

[15] Huang T., Xiong Y., Chen N., Wang D., Lai Y., Deng C. (2016). Highly selective enrichment of baicalin in rat plasma by boronic acid-functionalized core-shell magnetic microspheres: Validation and application to a pharmacokinetic study. Talanta.

[16] Lu C.M., Lin L.C., Tsai T.H. (2014). Determination and pharmacokinetic study of gentiopicroside, geniposide, baicalin, and swertiamarin in Chinese herbal formulae after oral administration in rats by LC-MS/MS. Molecules.

[17] Chen H., Li Z., Li Y.J., Wu X.W., Wang S.R., Chen K., Zheng X.X., Du Q., Tang D.Q. (2015). Simultaneous determination of baicalin, oroxylin A-7-*O*-glucuronide and wogonoside in rat plasma by UPLC-DAD and its application in pharmacokinetics of pure baicalin, Radix Scutellariae and Yinhuang granule. Biomed. Chromatogr..

[18] Cui X.B., Qian X.C., Huang P., Zhang Y.X., Li J.S., Yang G.M., Cai B.C. (2015). Simultaneous determination of ten flavonoids of crude and wine-processed Radix Scutellariae aqueous extracts in rat plasma by UPLC-ESI-MS/MS and its application to a comparative pharmacokinetic study. Biomed. Chromatogr..

[19] Shan W., Cheng J., Qu B., Sai J., Kong H., Qu H., Zhao Y., Wang Q. (2015). Development of a Fluorescence-Linked Immunosorbent Assay for Baicalin. J. Fluoresc..

[20] Hong Y., He W., Li D., He L., Zhang W. (2012). Preparation and *in vitro* Anti-tumor Effect of Baicalin Liposome. China J. Exp. Tradit. Med. Formula.

[21] Chen Y.J., Jia Y., Jin R., Xu J.Y., Xing X., Yuan J.L., Liu S.Z. (2011). Preparation of Baicalin Flexible nano Liposomes. China J. Exp. Tradit. Med. Formula.

[22] Li T., Zhang L., Tong L., Liao Q. (2014). High-throughput salting-out-assisted homogeneous liquid-liquid extraction with acetonitrile for determination of baicalin in rat plasma with high-performance liquid chromatography. Biomed. Chromatogr..

[23] Gao R., Zheng Q., Gong T., Fu Y., Deng L., Zhang Z.R. (2007). Gradient high-performance liquid chromatography for the simultaneous determination of chlorogenic acid and baicalin in plasma and its application in the study of pharmacokinetics in rats. J. Pharm. Biomed. Anal..

[24] Qiu F., He Z.G., Li H.Z. (2003). HPLC analyses and pharmacokinetic studies of baicalin and oxymat-rine in rabbits. Pharmazie.

[25] Xing J., Chen X., Zhong D. (2005). Stability of baicalin in biological fluids *in vitro*. J. Pharm. Biomed. Anal..

[26] Feng Z.Q., Han J., Xie Z.Y., Liao Q.F., Zhang L. (2012). Determination of plasma protein binding rate of multicomponent in S cutellaria baicalensis Georgi. Chin. Pharmacol. Bull..

[27] Kim Y.H., Jeong D.W., Paek I.B., Ji H.Y., Kim Y.C., Sohn D.H., Lee H.S. (2006). Liquid chrom-atography with tandem mass spectrometry for the simultaneous determinationof baicalein, b-aicalin, oroxylin A and wogonin in rat plasma. J. Chromatogr. B Anal. Technol. Biomed. Life Sci..

[28] Tong L., Wan M., Zhang L., Zhu Y., Sun H., Bi K. (2012). Simultaneous determination of baicalin, w-ogonoside, baicalein, wogonin, oroxylin A and chrysin of Radixscutellariae extract in rat pla-sma by liquid chromatography tandem mass spectrometry. J. Pharm. Biomed. Anal..

[29] Xiong X., Zhang L., Cheng L., Mao W. (2015). High-throughput salting-out assisted liquid-liquid extraction with acetonitrile for the determination of anandamide in plasma of hemodialysis patients with liquid chromatography tandem mass spectrometry. Biomed. Chromatogr..

[30] Wei Y., Zhao L. (2014). Passive lung targeted drug delivery systems via intravenous administration. Pharm. Dev. Technol..

[31] Waser P.G., Muller U., Kreuter J., Berger S., Munz K., Kaiser E., Pfluger B. (1987). Localization of colloidal particles (liposomes, hexylcyanoacrylate nanoparticles and albumin nanoparticles) by histology and autoradigraphy in mice. Int. J. Pharm..

